# Phase I/II study of adjuvant immunotherapy with sentinel lymph node T lymphocytes in patients with colorectal cancer

**DOI:** 10.1007/s00262-015-1715-3

**Published:** 2015-05-20

**Authors:** Yun-Huan Zhen, Xiao-Hui Liu, Yuan Yang, Bo Li, Jing-Ling Tang, Qiang-Xing Zeng, Jie Hu, Xing-Nan Zeng, Lu Zhang, Ze-Jun Wang, Xiao-Yun Li, Hui-Xin Ge, Ola Winqvist, Ping-Sheng Hu, Jin Xiu

**Affiliations:** Department of Colorectal Surgery, The Affiliated Hospital of Guiyang Medical College, Guiyang, People’s Republic of China; Cancer Immunology and Immunotherapy Center, The Affiliated Hospital of Guiyang Medical College, 28 Guiyi Street, Guiyang, 550004 Guizhou Province People’s Republic of China; Department of Gastrointestinal Surgery, The Affiliated Cancer Hospital of Guiyang Medical College, Guiyang, People’s Republic of China; Translational Immunology Unit, Department of Medicine, Karolinska University Hospital, Stockholm, Sweden; Research and Development, Sinorda Biomedicine, Guiyang, People’s Republic of China

**Keywords:** Colorectal cancer, Adoptive immunotherapy, Sentinel lymph node, T lymphocytes, Phase I/II study

## Abstract

Although the development of multi-disciplinary management has improved the survival of colorectal cancer (CRC), the prognosis of metastatic CRC patients remains poor. Accumulating evidence has demonstrated that immunotherapy with cancer vaccines and adoptive T cell transfusions may improve outcomes as an adjuvant to current standard CRC treatment. In this phase I/II study, 71 CRC patients who underwent radical surgery (stage I–III, *n* = 46) or palliative surgery (stage IV with non-resectable synchronous metastases, *n* = 25) were included. In the first part of this study, sentinel lymph nodes (SLNs) were intraoperatively identified in 55 patients (46 with stage I–III CRC and 9 with stage IV CRC). SLN-T lymphocytes were expanded ex vivo for a median of 28.5 days (range 23–33 days). Thereafter, a median of 153 × 10^6^ cells (range 20.7–639.0 × 10^6^) were transfused. No treatment-related toxicity was observed. In the second part of this study, the stage IV patients were routinely followed. The 24-month survival rate of the SLN-T lymphocyte group was significantly higher than that of the control group: 55.6 versus 17.5 % (*p* = 0.02). The median overall survival of the SLN-T lymphocyte and control groups was 28 and 14 months, respectively. Our study showed that adjuvant SLN-T lymphocyte immunotherapy is feasible and safe for postoperative CRC patients. Additionally, this therapy may improve the long-term survival of metastatic CRC. Further investigation of the clinical efficacy and anti-tumor immunity is warranted.

## Introduction

Colorectal cancer (CRC), one of the leading causes of cancer-related death, constitutes a major health problem worldwide. The CRC mortality rate varies by country and is affected by several factors, including the local incidence, the stage at diagnosis, the presence of factors associated with poor prognosis and the effectiveness of treatments [1]. With the development of multi-disciplinary treatment approaches and noninvasive screening, the 5-year survival rate of CRC improved from 58.0 to 64.9 % from 2003 to 2009 in the USA [2]. Although improved survival has been observed in many countries, a significant percentage (>20–25 %) of patients exhibit distant metastases at the time of diagnosis [3]. Among patients (stage I–III) who are eligible to receive curative resection, approximately 35 % develop tumor recurrence with eventual distant metastases during the disease course, especially within the first 3 years post-surgery [4]. The 5-year survival rate of non-resectable metastatic CRC (mCRC) is <12.5 % [2]. Therefore, there remains a significant need to effectively prevent tumor recurrence and to further improve the treatment outcomes for non-resectable mCRC.

During the last decade, cancer immunotherapy has emerged as a promising method of treating cancer [5, 6]. Growing clinical evidence has demonstrated that immune-based therapies are efficacious against certain types of cancer [7, 8]. In CRC, immunotherapy using cancer vaccines or adoptive T cell transfusion has exhibited promising therapeutic efficacy in prolonging progression-free survival and long-term survival, and immunotherapy may improve outcomes as an adjuvant to current standard treatment regimens [9–16].

Peripheral blood mononuclear cells (PBMCs) and tumor-infiltrating lymphocytes (TILs) are the most commonly reported starting materials for adoptive T cell immunotherapy. The expansion of cytotoxic T cells from tumor-draining lymph nodes (TDLNs) was first reported by Yanagawa [17]. Subsequent studies demonstrated that the TDLNs serve as a potential source of tumor-reactive T cells [15, 18–20]. To identify the optimal location to obtain tumor-reactive T cells for adoptive immunotherapy for CRC, tumor reactivity was compared between PBMCs, TILs and SLN-T cells from CRC patients [21]. SLN-T cells were found to represent an enriched source of tumor-reactive lymphocytes that proliferate upon stimulation with autologous tumor antigen.

In this phase I/II study, we evaluated the feasibility, toxicity and clinical effect of SLN-T cell-based adjuvant immunotherapy in postoperative CRC patients.

## Materials and methods

### Patients

Stage I–IV CRC patients were recruited at the Department of Colorectal Surgery of the Affiliated Hospital of Guiyang Medical College and the Department of Gastrointestinal Surgery of the Affiliated Cancer Hospital of Guiyang Medical College. Patients were selected according to the following criteria: age ≥18 years; histologically confirmed CRC with a life expectancy ≥3 months; patients (stage I–III) who underwent radical surgery; patients (stage IV) with synchronous metastases who underwent palliative surgery; and Eastern Cooperative Oncology Group (ECOG) performance status of 0–2. Patients were excluded if they had received neo-adjuvant chemo (radio) therapy before surgery, had a history of autoimmune disease or immunodeficiency syndrome, were currently treated with steroids or exhibited contraindications of leukapheresis.

The study protocol was reviewed and approved by the hospital ethical committee. All patients provided written informed consent before entering the study.

### Study design

This phase I/II study consisted of two parts. In the first part, the feasibility and safety of SLN-T cell transfusion in an adjuvant setting were studied. The SLNs were intraoperatively identified using patent blue injections. Patients who demonstrated successful ex vivo expansion of SLN-T cells received intravenous cell transfusion. Patients with (1) stage II (along with high-risk factors), (2) stage III or (3) stage IV disease received standard 5-fluorouracil (5-FU)-based chemotherapy within 8 weeks after surgery. The SLN-T cells were transfused 7 days after the first cycle of chemotherapy; the second phase of the study was designed to explore the potential efficacy of SLN-T cell immunotherapy as a supplement to standard chemotherapy in stage IV patients after palliative surgery. The OS of these patients was followed from the day of enrollment until death.

### Intraoperative identification of SLNs

The SLNs were identified as described previously [22]. In brief, 1 ml of patent blue dye was injected under the serosa surrounding the primary tumor. Within 5 min, the SLN stained blue. The SLN was excised and cut in half; one half was subjected to flow cytometry analysis and ex vivo expansion, and the remaining half was used for routine histopathological examination.

### Immunological evaluation of SLN-derived lymphocytes

Single-cell suspensions from SLNs and tumor tissue were obtained immediately after surgery by applying gentle pressure using a loose-fit glass homogenizer as described by Marits [21]. PBMCs were purified by Ficoll-Paque (Amersham). For phenotypic analysis of the lymphocytes from SLNs, TILs and PBMCs, fluorescent-labeled monoclonal antibodies (mAbs) against CD45, CD3, CD4, CD8, CD16, CD56, CD19, CD69, CD25, CD127, CD45RA and CCR7 (Beckman Coulter) were used. Cells were incubated in the presence of mAbs according to the manufacturer’s recommendations for 20 min at room temperature (18–25 °C) and protected from light. After incubation, the cell suspensions were washed with phosphate-buffered saline (PBS), and the cell pellets were resuspended in 0.5 ml of PBS for analysis. Samples were further analyzed using a FC500 flow cytometer (Beckman Coulter). At least 50,000 total events were collected and analyzed using CXP software (Beckman Coulter).

An enzyme-linked immunospot (ELISPOT) assay kit (Mabtech AB, Sweden) was used to evaluate the antigen-specific T cells by measuring the release of interferon gamma (IFN-γ). Lymphocytes (1 × 10^5^/well) isolated from SLNs and PBMCs were seeded in 96-well plates that were pre-coated with an anti-human IFN-γ antibody in triplicate and incubated with autologous tumor lysates or an anti-CD3 mAb. After 48 h of incubation, the assay was developed according to the standard protocol. The membranes were air-dried, and the spots in each well were subjected to automated evaluation using the AID FluoroSpot Reader System (Autoimmun Diagnostika GmbH, Germany).

### Ex vivo expansion of SLN-T cells

Single-cell suspensions obtained from SLNs were resuspended in X-VIVO™ 15 serum-free cell culture medium (LONZA) at a density of 4 × 10^6^ cells/ml in the presence of 1000 IU/ml recombinant human interleukin-2 (Shuanglu, China). These cells were plated in flasks or plates and maintained in a humidified atmosphere containing 5 % CO_2_ at 37 °C. The autologous tumor lysate was added to the initial culture at a dilution of 1/100 (v/v) as described previously [15]. To induce highly tumor-specific SLN-T cells, re-stimulation was performed by adding autologous tumor lysate together with irradiated autologous PBMCs during SLN-T cell cultures. One week before transfusion, 5 ml of culture medium was removed for a bacterial and fungal contamination test using BACTEC 9120 (Becton–Dickinson), and the endotoxin levels were measured based on the Limulus reaction. On the day of transfusion, these assays were repeated to detect any bacterial, fungal or endotoxin contamination. The lymphocyte subsets of SLN-T cells were analyzed. Furthermore, 1 × 10^6^ cells were used for flow cytometry analysis of the tumor surface marker epithelial cell adhesion molecule (EpCAM) to exclude the presence of tumor cells.

### Adoptive transfusion of SLN-T cells and toxicity evaluation

The final SLN-T cells were harvested, washed twice in saline solution and transferred to a sterile plastic bag containing 200 ml of saline solution and 1 % human serum albumin (CSL Behring GmbH, Germany). The cells were intravenously transfused over a 60-min interval according to the blood transfusion guidelines of the hospital. Transfusion-related toxicity was assessed post-cell transfusion using the Common Terminology Criteria for Adverse Events (CTCAE) 3.0 criteria.

### Follow-up of stage IV patients

The stage IV CRC patients were followed every 3 months in the first year and every 6 months beginning in the second year. The disease status was assessed based on physical examination, the serum level of carcinoma embryonic antigen (CEA), chest CT, abdominal CT and colonoscopy.

### Statistical analysis

Statistical analysis was performed using GraphPad Prism version 5.0 (GraphPad Software, Inc, San Diego, CA). Changes in surface markers and INF-γ release between the groups were assessed using Student’s *t* test or one-way ANOVA. Categorical variables were compared using the Chi-squared and Fisher’s exact tests. Kaplan–Meier curves were used to assess the influence of SLN-T cell immunotherapy on OS. The significance of the difference between two groups was assessed using the log-rank test. All results were considered to be significant at *p* < 0.05.

## Results

### Patient characteristics

**Fig. 1:**
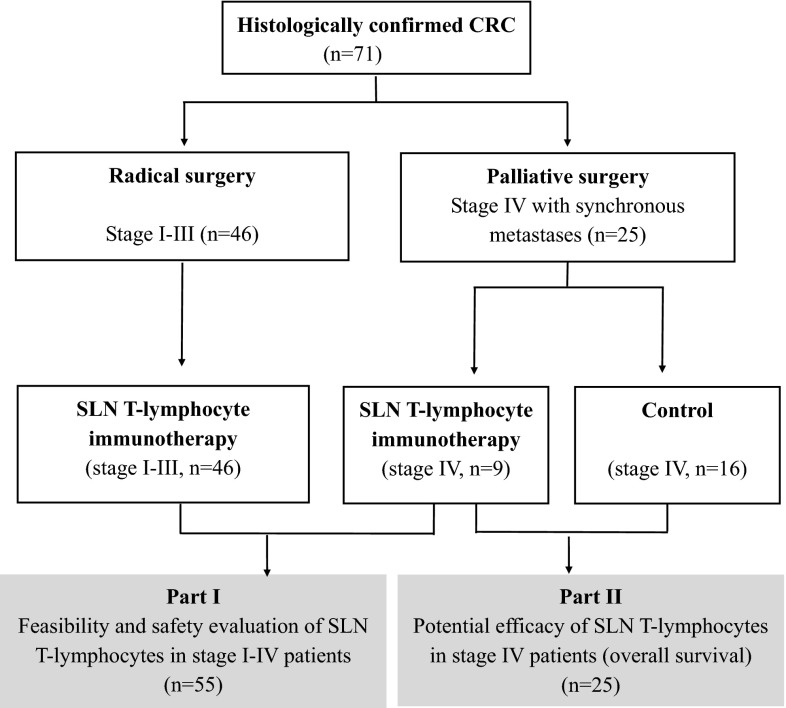
Study design. Patients with stage I–III CRC undergoing radical surgery and patients with stage IV CRC undergoing palliative resection were included in this study. In part I, the feasibility and safety of SLN-T cell as an adjuvant treatment were evaluated in 55 postoperative stage I–IV patients. In part II, the efficacy of SLN-T cell treatment was further evaluated by measuring overall survival (OS) in 25 stage IV CRC patients

Eighty-seven CRC patients were enrolled between June 2010 and August 2013. Among them, 71 patients (stage I–IV) were included in the SLN-T immunotherapy group, and the remaining 16 stage IV patients, who received palliative surgery and standard chemotherapy, served as controls. Because of the unsuccessful expansion of SLN-T cells from 16 patients in the SLN-T immunotherapy group, 55 patients (46 stage I–III and 9 stage IV, as shown in Table 1) received SLN-T cell transfusion. Together with the 16 stage IV CRC patients in the control group, a final total number of 71 CRC patients (46 stage I–III and 25 stage IV) were included for analysis in this study (Fig. 1).

**Table 1:** Characteristics of patients who received SLN-T cell transfusion

Patient	Stage I	Stage II	Stage III	Stage IV
Number (*n* = 55)	5	20	21	9
Gender				
M	3	16	13	5
F	2	4	8	4
Age (median, years)	65 (62–69)	60.5 (37–76)	58.8 (32–73)	56 (45–74)
Primary sites of disease				
Colon	2	13	11	7
Rectum	3	7	10	2

The characteristics of the 25 stage IV CRC patients in the control group (*n* = 16) and the SLN-T immunotherapy group (*n* = 9) are summarized in Table 2. The SLN-T and control groups did not significantly differ with respect to age, sex, histological grade, the characteristics of the primary tumor or the lymph nodes, distant metastasis, the levels of CEA, hemoglobin, alkaline phosphatase (ALP), bilirubin, creatinine, albumin, alanine aminotransferase (ALT) or aspartate transaminase (AST), surgical intervention for the primary and metastatic tumors or the number of cycles of chemotherapy received post-operation. Six patients (37.5 %) in the control group received second-line treatment with an anti-EGFR/VEGF-targeted agent, whereas none of the patients in the SLN-T group received this treatment (*p* = 0.06).

**Table 2:** Baseline characteristics of stage IV CRC patients

Characteristic	Control (*n* = 16)	SLN-T (*n* = 9)	Total (*n* = 25)	*p*
Median age (range), years	51 (32.0–76.0)	56.0 (45.0–74.0)	55.0 (32.0–76.0)	0.205
Sex				
Female	3 (18.8)	4 (44.4)	7 (28.0)	0.21
Male	13 (81.2)	5 (55.6)	18 (72.0)	
Primary sites of disease				
Colon	10 (62.5)	7 (77.8)	17 (68.0)	0.66
Rectum	6 (37.5)	2 (22.2)	8 (32.0)	
Histology				
High (grade 3–4)	11 (68.8)	6 (66.7)	17 (68.0)	1.00
Low (grade 1–2)	5 (31.2)	3 (33.3)	8 (32.0)	
Primary tumor (T)				
T1/T2	0 (0.0)	0 (0.0)	0 (0.0)	0.235
T3	9 (56.3)	8 (88.9)	17 (68.0)	
T4	6 (37.5)	1 (11.1)	7 (28.0)	
Tx	1 (6.2)	0 (0.0)	1 (4.0)	
Lymph node involvement (*N*)				
N0	2 (12.5)	3 (33.3)	5 (20.0)	0.09
N1	6 (37.5)	4 (44.4)	10 (40.0)	
N2	1 (6.3)	2 (22.2)	3 (12.0)	
Nx	7 (43.7)	0 (0.0)	7 (28.0)	
Distant metastasis (*M*)				
M1a	10 (62.5)	2 (22.2)	12 (48.0)	0.97
M1b	6 (37.5)	7 (77.8)	13 (52.0)	
CEA				
Normal	2 (12.5)	3 (33.3)	5 (20.0)	0.31
Abnormal	14 (87.5)	6 (66.7)	20 (80.0)	
Hemoglobin				
Normal	7 (43.8)	6 (66.7)	13 (52.0)	0.41
Abnormal	9 (56.2)	3 (33.3)	12 (48.0)	
ALP				
Normal	13 (81.3)	8 (88.9)	21 (84.0)	1.00
Abnormal	3 (18.7)	1 (11.1)	4 (16.0)	
Bilirubin				
Normal	14 (87.5)	9 (100.0)	23 (92.0)	0.52
Abnormal	2 (12.5)	0 (0.0)	2 (8.0)	
Creatinine				
Normal	13 (81.3)	8 (88.9)	21 (84.0)	1.00
Abnormal	3 (18.7)	1 (11.1)	4 (16.0)	
Albumin				
Normal	12 (75.0)	8 (88.9)	20 (80.0)	0.62
Abnormal	4 (25.0)	1 (11.1)	5 (20.0)	
ALT				
Normal	13 (81.3)	7 (77.8)	20 (80.0)	1.00
Abnormal	3 (18.7)	2 (22.2)	5 (20.0)	
AST				
Normal	14 (87.5)	9 (100.0)	23 (92.0)	0.52
Abnormal	2 (12.5)	0 (0.0)	2 (8.0)	
Surgical management				
Primary tumor resection	9 (56.3)	9 (100.0)	18 (72.0)	
Metastatic tumor resection	0 (0.0)	0 (0.0)	0 (0.0)	
Cycles of chemo				
<4	8 (50.0)	4 (44.4)	12 (48.0)	1.00
≥4	8 (50.0)	5 (55.6)	13 (52.0)	
Anti-EGFR/VEGF therapy				
Yes	6 (37.5)	0 (0.0)	6 (24.0)	0.06
No	10 (62.5)	9 (100.0)	19 (76.0)	

*ALP* alkaline phosphatase, *ALT* alanine aminotransferase, *AST* aspartate transaminase, *CEA* carcinoembryonic antigen

### SLN identification and phenotypic analysis of lymphocyte subtypes

**Fig. 2:**
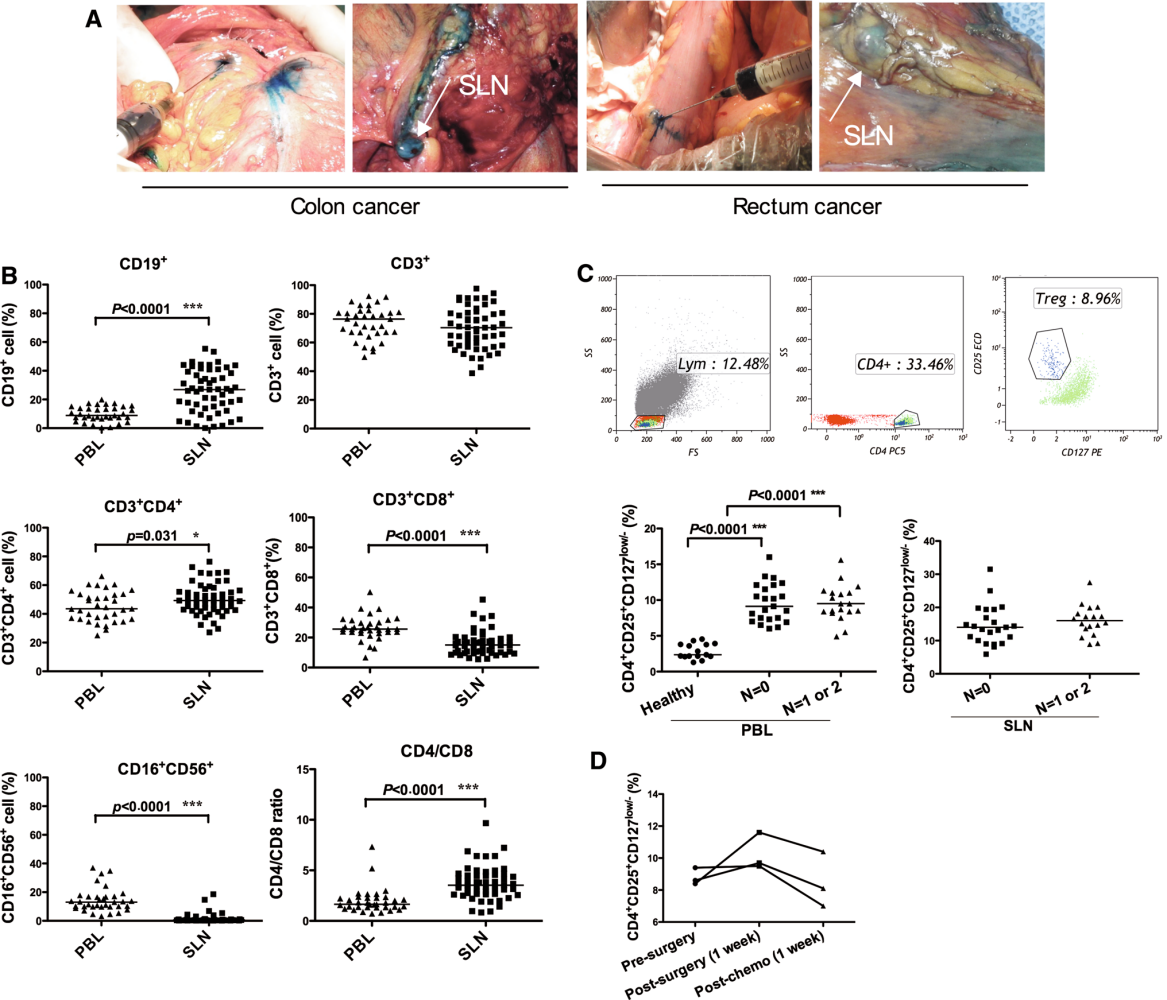
Identification of SLN and lymphocyte populations in the SLN and corresponding PBL of CRC patients. **a** Within 5 min of intraoperative injection of patent *blue dye* around the primary tumor, the SLN *stained blue*. **b** The proportion of CD19^+^, CD3^+^, CD3^+^CD4^+^, CD3^+^CD8^+^, CD16^+^CD56^+^ cells, and the CD4/CD8 ratio in SLN and corresponding PBL. **c** The proportion of CD4^+^CD25^hi^CD127^low/−^ Tregs in PBL and SLNs of patients with tumor-free lymph node (*N* = 0) and those with metastatic lymph node (*N* = 1 or 2) determined by flow cytometry. **d** The changes of CD4^+^CD25^hi^CD127^low/−^ Tregs in PBL of stage III CRC patients (*n* = 3) after surgery and systematic 5-FU-based chemotherapy

The SLNs were intraoperatively identified by injecting patent blue in the circumference of the tumor (Fig. 2a). One to three identified SLNs were collected into a 15-ml tube containing pre-chilled X-VIVO™ 15 medium. The lymphocyte subtypes present in the SLN and corresponding peripheral blood (PBL) were determined by flow cytometry. A significant difference in lymphocyte composition was observed between the SLNs and the PBL. The proportions of CD19^+^ B lymphocytes (*p* < 0.0001) and CD3^+^CD4^+^ T lymphocytes (*p* = 0.031) were markedly higher in SLNs, whereas the cytotoxic CD3^+^CD8^+^ T lymphocytes (*p* < 0.0001) and CD16^+^CD56^+^ natural killer (NK) cells (*p* < 0.0001) were significantly less prevalent in SLNs. As a consequence of the increased proportion of CD3^+^CD4^+^ T lymphocytes and the decreased proportion of CD3^+^CD8^+^ T lymphocytes, the CD4/CD8 ratio was dramatically higher in SLNs than in the PBL (*p* < 0.0001) (Fig. 2b).

Given the elevated proportion of CD3^+^CD4^+^ T lymphocytes observed in SLNs, we further examined the presence of regulatory T cells (Tregs) in the SLNs and corresponding PBL by identifying the CD4^+^CD25^hi^CD127^low/−^ cells (Fig. 2c). The proportion of circulating CD4^+^CD25^hi^CD127^low/−^ Tregs in the PBL was significantly higher in CRC patients than in healthy controls (*p* < 0.0001). The distribution of circulating Tregs in the PBL or in SLNs did not significantly differ between patients with tumor-free lymph nodes (N0) and those with metastatic lymph nodes (N1 or N2) (Fig. 2c).

### SLNs contain tumor-reactive T cells

**Fig. 3:**
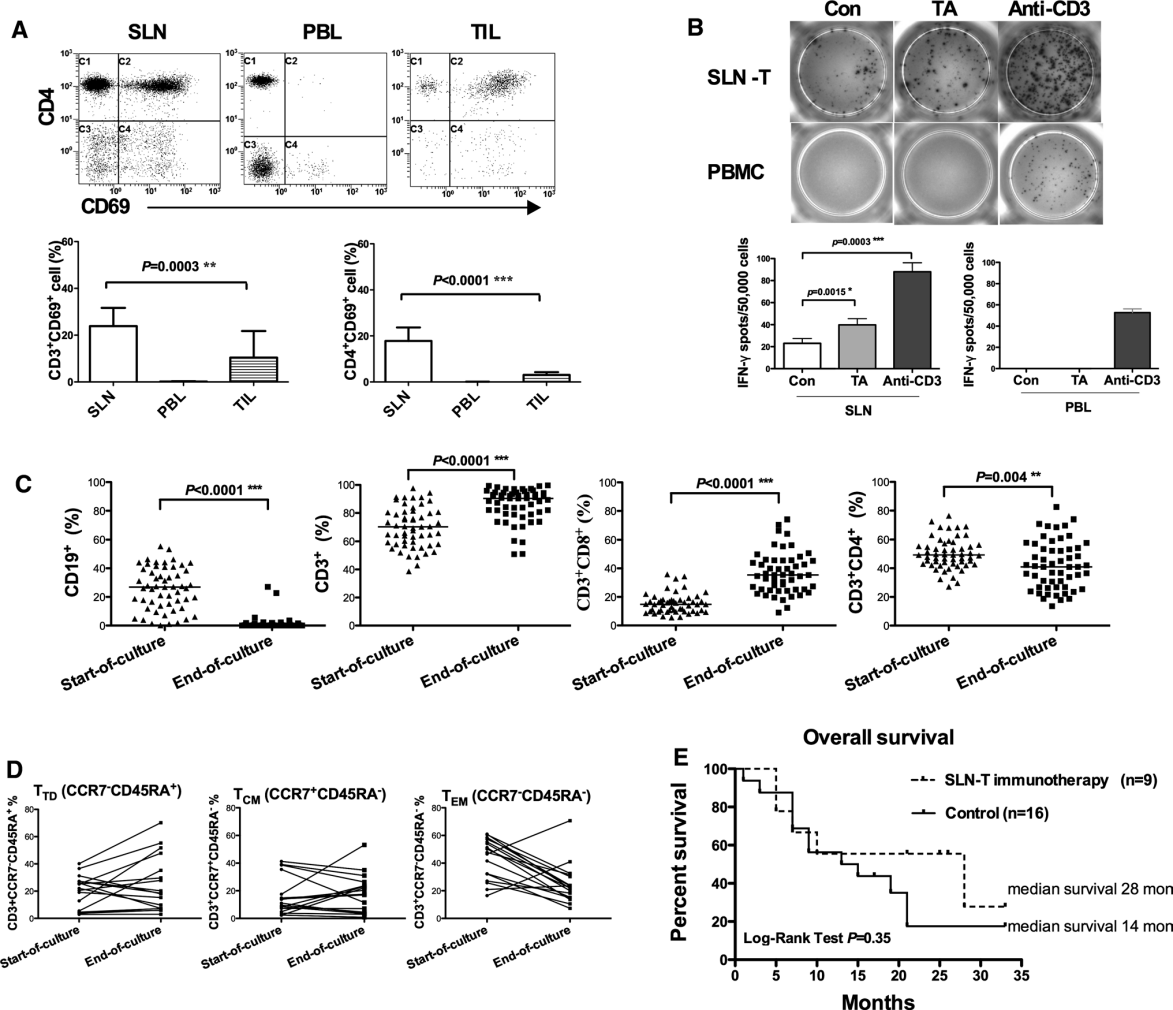
Phenotype and functional activity of ex vivo-expanded SLN-T cells and the Kaplan–Meier curves of stage IV patients. **a** The proportion of CD3^+^CD69^+^, CD4^+^CD69^+^T lymphocytes in SLNs, PBL and TILs. **b** IFN-γ ELISPOT assays were performed to determine the tumor-specific response of SLN lymphocytes and corresponding PBL lymphocytes. **c** Ex vivo expansion of SLN-T cells resulted in polyclonal expansion of CD3^+^CD8^+^ and CD3^+^CD4^+^ cells and a diminished CD19^+^ B cell population. **d** At the end of ex vivo expansion, the memory phenotype with CCR7 and CD45RA was characterized by flow cytometry (*n* = 17). **e** Kaplan–Meier curves for 33-month survival of stage IV CRC patients

To evaluate the activation status of lymphocytes in SLNs, TILs and PBL, the expression of the very early activation marker CD69 was investigated [21]. The SLNs contained a significantly higher proportion of activated CD3^+^CD69^+^ (*p* = 0.0003) and CD4^+^CD69^+^ (*p* < 0.0001) T lymphocytes compared to TILs. In the PBL, no activation of T lymphocytes was detected (Fig. 3a).

To evaluate the tumor-specific responses of the SLN lymphocytes to autologous tumor lysate, we performed IFN-γ ELISPOT assays (Fig. 3b). Significantly enhanced IFN-γ release in response to autologous tumor antigens was observed in SLN-T lymphocytes (*p* = 0.0015). Interestingly, harvested SLNs not treated with autologous tumor antigen showed a low level of spontaneous IFN-γ secretion, suggesting the initiation of T lymphocyte activation in the SLN. However, in the PBL, no tumor-reactive IFN-γ-secreting lymphocytes were observed when stimulated with autologous tumor antigens (Fig. 3b).

### Profiles of surface markers in ex vivo-expanded SLN-T cells

*Ex vivo* expansion of SLN-T cells was performed for a median of 28.5 days (range 23–33 days) in culture. The cultures of SLN-T cells proliferated in response to autologous tumor antigens, and the median cell number reached 153.0 × 10^6^ (range 20.7–639.0 × 10^6^) for transfusion.

A comparison of surface marker expression between the starting culture and the ending culture (Fig. 3c) revealed that the percentage of CD19^+^ B lymphocytes significantly decreased from 25.8 ± 14.9 % to 1.5 ± 4.7 % (*p* < 0.0001). Simultaneously, a substantial increase in the percentage of CD3^+^ T cells from 69.9 ± 14.2 % to 86.0 ± 11.8 % (*p* < 0.0001) was observed after ex vivo expansion. The expansion protocol resulted in alterations in the percentages of CD3^+^CD8^+^ cytotoxic T lymphocytes from 15.4 ± 6.9 % to 36.7 ± 14.6 % (*p* < 0.0001), CD3^+^CD4^+^ lymphocytes from 50.1 ± 10.7 % to 42.2 ± 16.9 % (*p* = 0.004) and CD16^+^CD56^+^ NK cells from 1.5 ± 3.3 % to 12.0 ± 10.6 % (*p* < 0.0001) (data not shown). We next determined the memory phenotype using the lymph node-homing chemokine receptor marker CCR7 together with CD45RA to further characterize the phenotype and the function of tumor-specific SLN-T cells. At the end of ex vivo expansion, the majority of the cultured cells exhibited a differentiated effector T (CCR7^−^CD45RA^+^) or central memory T (CCR7^+^CD45RA^−^) phenotype, whereas the proportion of the effector memory T (CCR7^−^CD45RA^−^) subpopulation had dramatically decreased (Fig. 3d). This result demonstrated the predominant expansion of activated tumor-specific effector and central memory T cells.

### Treatment-related toxicity in stage I–IV patients and survival of stage IV patients

The SLN-T cell transfusion-related toxicity in 55 patients (46 stage I–III and 9 stage IV) is summarized in Table 3. No significant induction of toxicity was observed after intravenous administration of SLN-T cells.

**Table 3:** SLN-T cell transfusion-related toxicity evaluated by CTCAE version 3.0

Grade of adverse events	Pre (*n* = 55)				Post (*n* = 55)			
	Grade 1 *n* (%)	Grade 2 *n* (%)	Grade 3 *n* (%)	Grade 4 *n* (%)	Grade 1 *n* (%)	Grade 2 *n* (%)	Grade 3 *n* (%)	Grade 4 *n* (%)
Albumin	9 (16.4)	0	0	0	3 (5.5)	0	0	0
ALT	12 (21.8)	1 (1.8)	0	0	15 (27.3)	0	0	0
AST	15 (27.3)	0	0	0	5 (9.1)	0	0	0
Bilirubin	2 (3.6)	0	0	0	2 (3.6)	0	0	0
Creatinine	54 (98.2)	1 (1.8)	0	0	54 (98.2)	1 (1.8)	0	0
Hemoglobin	20 (36.4)	8 (14.5)	4 (7.3)	0	25 (45.5)	9 (16.4)	1 (1.8)	0
Leukocytes	8 (14.5)	3 (5.5)	0	0	10 (18.2)	1 (1.8)	0	0
Lymphopenia	8 (14.5)	4 (7.3)	0	0	3 (5.5)	2 (3.6)	0	0
Neutrophils	12 (21.8)	8 (14.5)	0	0	7 (12.7)	4 (7.3)	0	0
Platelets	2 (3.6)	0	0	0	3 (5.5)	0	0	0
Allergy	0	0	0	0	0	0	0	0
Nausea	0	0	0	0	0	0	0	0
Vomiting	0	0	0	0	0	0	0	0
Diarrhea	0	0	0	0	0	0	0	0

*ALT* alanine aminotransferase, *AST* aspartate transaminase

Because of their palliative situation, the stage IV patients in both the SLN-T immunotherapy and control groups received treatments deemed necessary to provide adequate supportive care. During follow-up, the patients exhibiting disease progression received second-line chemotherapy with or without anti-EGFR/VEGF-targeted therapy. Furthermore, patients exhibiting synchronized liver metastasis were permitted to undergo radiofrequency ablation (RFA) and transcatheter arterial chemoembolization (TACE).

The 25 stage IV patients were followed for 33 months. We found a significantly increased 24-month survival rate in the SLN-T cell immunotherapy group compared to the control group: 55.6 vs. 17.5 % (*p* = 0.02). A tendency of an increased OS was observed among patients receiving SLN-T cell transfusion (*n* = 9, median OS 28 months) compared with the control (*n* = 16, median OS 14 months), although the log-rank test did not indicate a significant difference (*p* = 0.35) (Fig. 3e).

## Discussion

In this study, we demonstrate that SLNs are a naturally enriched source of tumor-reactive T cells that can be primed using autologous tumor antigens without prior in vivo vaccination or supplementation of antigens from synthesized tumor peptides or tumor cell lines. After polyclonal expansion of tumor-specific CD8^+^ and CD4^+^ cells, the SLN-T cells can be safely transfused back into patients as an adjuvant to current standard treatment regimens for CRC. Our results indicate the promising potential for developing SLN-T cell-based immunotherapy for CRC patients, especially for the improvement of the long-term survival of patients with non-resectable mCRC.

In previous studies, several groups reported in animal models that the injection of primary tumor cells induces sensitized T cells within the draining lymph nodes local to the injection sites. Tumor-reactive T cells from lymph nodes can be further expanded ex vivo in the presence of an anti-CD3 stimulus with or without CD28 engagement in addition to interleukin-2 to treat established tumors [17, 18, 23–27]. These preclinical studies were further verified in two feasibility studies and one phase II study of head and neck carcinoma [28], renal cell carcinoma [29, 30] and melanoma [30]. The results of these studies indicate that this approach induces a tumor-specific immune response in the draining lymph node, which is a feasible source of tumor-reactive T cells for effective immunotherapy. In our study, we demonstrate that SLNs naturally contain many more tumor-reactive T cells than the PBL and TILs. Without any prior tumor vaccination or additional invasive surgical procedures to obtain the enlarged vaccinated lymph node, the SLN-T cell population can be expanded reproducibly for immunotherapeutic purposes. The SLN is a specialized site at which lymphocytes first encounter tumor antigens in association with antigen-presenting cells (APCs) and initiate local anti-tumor immune responses. We demonstrate that the SLN represents a unique immune microenvironment in terms of the lymphocyte population, the lymphocyte activation status and the response of lymphocytes to autologous tumor antigens. Compared with the corresponding PBL or TILs, in the SLNs, the proportions of B lymphocytes, CD3^+^CD4^+^ and CD4^+^CD69^+^ T lymphocytes, together with the CD4^+^/CD8^+^ ratio, are significantly higher; this result reflects the preferable selection of T helper cells and B lymphocytes by the SLN microenvironment to enhance tumor antigen-presenting activity and anti-tumor immune regulation. Moreover, rather than repeatedly providing non-specific stimulation with anti-CD3/CD28, in our serum-free culture system, specific stimulation with autologous tumor antigen is performed during the initial and middle phases of culture, resulting in tumor-specific polyclonal expansion of CD8^+^ and CD4^+^ cells.

Tregs are key mediators in maintaining peripheral tolerance and inhibiting anti-tumor immune response within the tumor microenvironment. In previous studies, the correlation of the number of CD4^+^CD25^+^Foxp3^+^ Tregs in TDLNs with tumor stage and survival was contradictory [31, 32]. The transcription factor Foxp3 has been shown to play a crucial role in Treg development, but it is not strictly expressed by natural or induced Tregs. In this study, staining for the specific surface marker profile CD4^+^CD25^+^CD127^low/−^ was used to identify Tregs [33, 34]. We observed a significant elevation of the circulating CD4^+^CD25^+^CD127^low/−^ Treg population in CRC patients compared with healthy controls. In both PBL and the SLNs, the CD4^+^CD25^+^CD127^low/−^ Treg levels did not correlate with the disease severity. Moreover, we examined the circulating CD4^+^CD25^+^CD127^low/−^ Treg levels at specific time points after surgery and systemic adjuvant chemotherapy in three patients with stage III disease. The circulating CD4^+^CD25^+^CD127^low/−^ Treg levels tended to decline post-chemotherapy (Fig. 2d). This finding indicates that 5-FU-based cytotoxic chemotherapy can modulate the tumor microenvironment to augment anti-tumor immune responses. Therefore, a combinatorial approach of chemotherapy together with immunotherapy is recommended for future studies.

The current successful immunotherapies for cancer fall into several broad categories: (1) immune checkpoint blockade against inhibitory pathways targeting cytotoxic T lymphocyte-associated antigen-4 (CLTA-4) and programmed cell death protein 1 (PD-1) and its ligand (PD-L1) [35–37]; (2) cancer vaccines including autologous tumor cells, dendritic cells pulsed with specific tumor antigens [38] and several identified tumor peptide antigens [39, 40]; and (3) adoptive cell transfusion (ACT), including that of ex vivo-activated and ex vivo-expanded autologous T cells [41, 42] or genetically engineered T cells expressing chimeric antigen receptors [43, 44]. The vaccine and ACT approaches are the most typically explored immunotherapies for CRC. Among these approaches, the best-studied tumor vaccine, OncoVAX, consists of irradiated autologous tumor cells together with the adjuvant BCG. When tested in postoperative CRC patients, improvements in recurrence-free duration and survival were only observed in stage II patients [12]. The most successful ACT-based immunotherapy for solid tumors is the transfusion of TILs in metastatic melanoma patients [42, 45] together with lymph-depletion conditioning and administration of IL-2; however, the application of TILs to CRC is limited because of the absence of tumor-specific effector cells after ex vivo expansion [46]. Therefore, there is a need for a more effective immunotherapy for CRC. In our study, we demonstrate that SLN-T cell-based immunotherapy is feasible for stage I–IV CRC patients. Moreover, the transfusion of SLN-T cells at doses of up to 639.0 million cells is safe. Evaluating the clinical efficacy of SLN-T immunotherapy for stage IV patients enables the assessment of the survival benefits over a limited period. Our results demonstrate that SLN-T cell treatment significantly improved the survival rate of stage IV patients. Furthermore, a prolonged median OS was observed in the SLN-T immunotherapy group compared with the control group (28 vs. 14 months). However, because of the small sample size of this study, statistical significance was not reached. Further studies with larger sample sizes are warranted to more precisely determine the survival effect of SLN-T cell-based immunotherapy. Moreover, in this study, SLN-T cells have been administered in the absence of supporting adjuvants or cytokines that may enhance the in vivo persistence of transferred T cells. This needs to be investigated in future studies. In clinical practice, most stage IV CRC patients are not candidates for surgical resection, eliminating easy access to SLNs and autologous tumor cells. Therefore, patients who undergo curative resection of hepatic metastases represent the targeted patients for our next study. The potential benefits of resection of primary tumors in patients with stage IV CRC have been extensively studied [47]. Although the results are contradictory, the benefits of primary tumor resection should be further addressed. Immunotherapy should be added to current therapies to optimize clinical outcomes under the conditions of a minimal tumor burden. Moreover, the clinically beneficial effects of reducing tumor recurrence after surgery must be addressed in stage I–III CRC patients.

As a deeper understanding of the human immune system and tumor immunology has become available, successful immunotherapies against select tumor types have continued to be developed. Here, we demonstrate a phase I/II study of SLN-T cell-based adjuvant immunotherapy in 71 stage I–IV postoperative CRC patients. Our results demonstrate that SLN-T cell-based immunotherapy is feasible as an adjuvant to the current standard treatment regimens for CRC. At the dose range of 20.7–639.0 × 10^6^ total SLN-T cells, this treatment is safe. Moreover, this treatment may improve the long-term survival of non-resectable mCRC patients. Future investigations, including randomized control trials, are needed to confirm the survival benefit of this immunotherapy to stage IV CRC patients and to explore its possible efficacy in reducing the rate of recurrence in stage I–III CRC patients.

## Abbreviations

ACT: Adoptive cell transfusion
ALP: Alkaline phosphatase
ALT: Alanine aminotransferase
APC: Antigen-presenting cell
AST: Aspartate transaminase
CEA: Carcinoma embryonic antigen
CLTA-4: Cytotoxic T lymphocyte-associated antigen-4
CRC: Colorectal cancer
ECOG: Eastern Cooperative Oncology Group
ELISPOT: Enzyme-linked immunospot
EpCAM: Epithelial cell adhesion molecule
5-FU: 5-Fluorouracil
IFN-γ: Interferon gamma
mAbs: Monoclonal antibodies
mCRC: Metastatic colorectal cancer
NK: Natural killer
OS: Overall survival
PBL: Peripheral blood
PBMCs: Peripheral blood mononuclear cells
PD-1: Programmed cell death protein 1
RFA: Radiofrequency ablation
SLNs: Sentinel lymph nodes
TACE: Transcatheter arterial chemoembolization
T_CM_: Central memory T cell
TDLNs: Tumor-draining lymph nodes
T_EM_: Effector memory T cell
TILs: Tumor-infiltrating lymphocytes
Tregs: Regulatory T cells
T_TD_: Terminally differentiated T cell
